# 

**Published:** 1865-11

**Authors:** 


### Dental Register

### OF THE WEST.

EDITED AND PUBLISHED BY J. TAFT.

NOVEMBER, 1865.

MONTHLY:

*Three Dollars per Annum, in Advance.*

CINCINNATI:

WRIGHTSON & CO., Printers, 167 WALNUT STREET.

*J. & C. R. TAFT, Dentists, 172 Race Street.*

